# Robotic Modules for the Programmable Chemputation of Molecules and Materials

**DOI:** 10.1021/acscentsci.3c00304

**Published:** 2023-07-26

**Authors:** Daniel Salley, J. Sebastián Manzano, Philip J. Kitson, Leroy Cronin

**Affiliations:** School of Chemistry, University of Glasgow, University Avenue, Glasgow G12 8QQ, U.K.

## Abstract

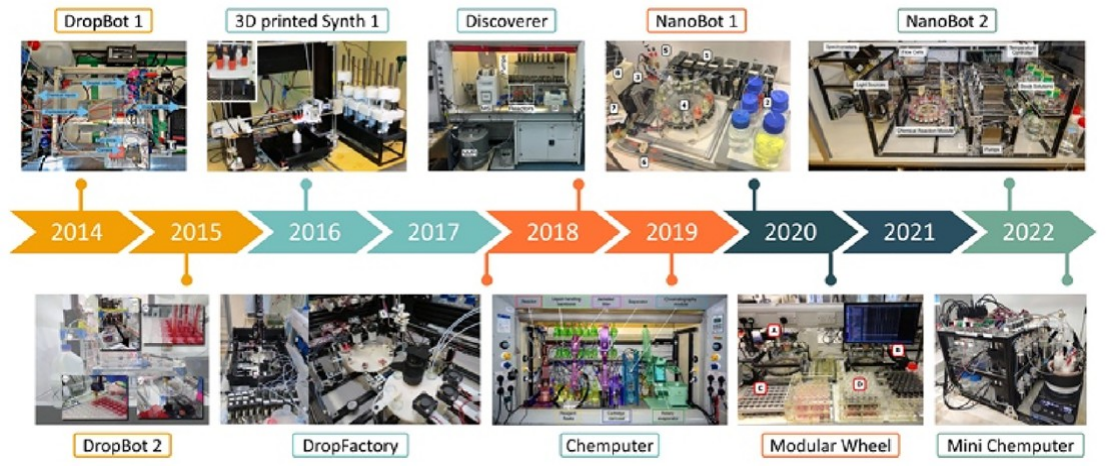

Before leveraging big data methods like machine learning and artificial intelligence (AI) in chemistry, there is an imperative need for an affordable, universal digitization standard. This mirrors the foundational requisites of the digital revolution, which demanded standard architectures with precise specifications. Recently, we have developed automated platforms tailored for chemical AI-driven exploration, including the synthesis of molecules, materials, nanomaterials, and formulations. Our focus has been on designing and constructing affordable standard hardware and software modules that serve as a blueprint for chemistry digitization across varied fields. Our platforms can be categorized into four types based on their applications: (i) discovery systems for the exploration of chemical space and novel reactivity, (ii) systems for the synthesis and manufacture of fine chemicals, (iii) platforms for formulation discovery and exploration, and (iv) systems for materials discovery and synthesis. We also highlight the convergent evolution of these platforms through shared hardware, firmware, and software alongside the creation of a unique programming language for chemical and material systems. This programming approach is essential for reliable synthesis, designing experiments, discovery, optimization, and establishing new collaboration standards. Furthermore, it is crucial for verifying literature findings, enhancing experimental outcome reliability, and fostering collaboration and sharing of unsuccessful experiments across different research labs.

## Abstract 2

We provide an account of the development and use of a suite of automated platforms, and a universal digitization standard, for chemistry. We detail the requirements which drove this development.

## Introduction

Many areas of experimental chemistry and materials science have always been tedious, time-consuming, and in many cases irreproducible.^1^ As our need for rapid and efficient exploration of the chemical and materials space has evolved, the automation of repetitive and difficult tasks has become a priority, and this has resulted in the development of several commercially available systems. Many of these systems were designed for specific sets of chemistry (e.g., peptides synthesis,^2^ oligossacharides,^3^ oligonucleotides^4^), but more complex platforms such as Chemspeed^5^ and Labman have also become available for a wider range of applications. These platforms have become common fixtures in laboratory settings, performing their intended tasks with great success and unmatched reproducibility. However, commercial platforms can be expensive, require highly trained personnel, and may not be able to provide a standard for reporting work due to the lack of shared software with little interoperability. These issues have ignited the need for the development of custom-made platforms that can perform complex and high throughput procedures both in industry^6,7^ and academia.^8−14^ More recently, the emergence of open-source hardware and software, rapid additive manufacturing techniques, and increased access to general engineering equipment have opened the possibility to develop low-cost, bespoke platforms.

**Figure 1:**
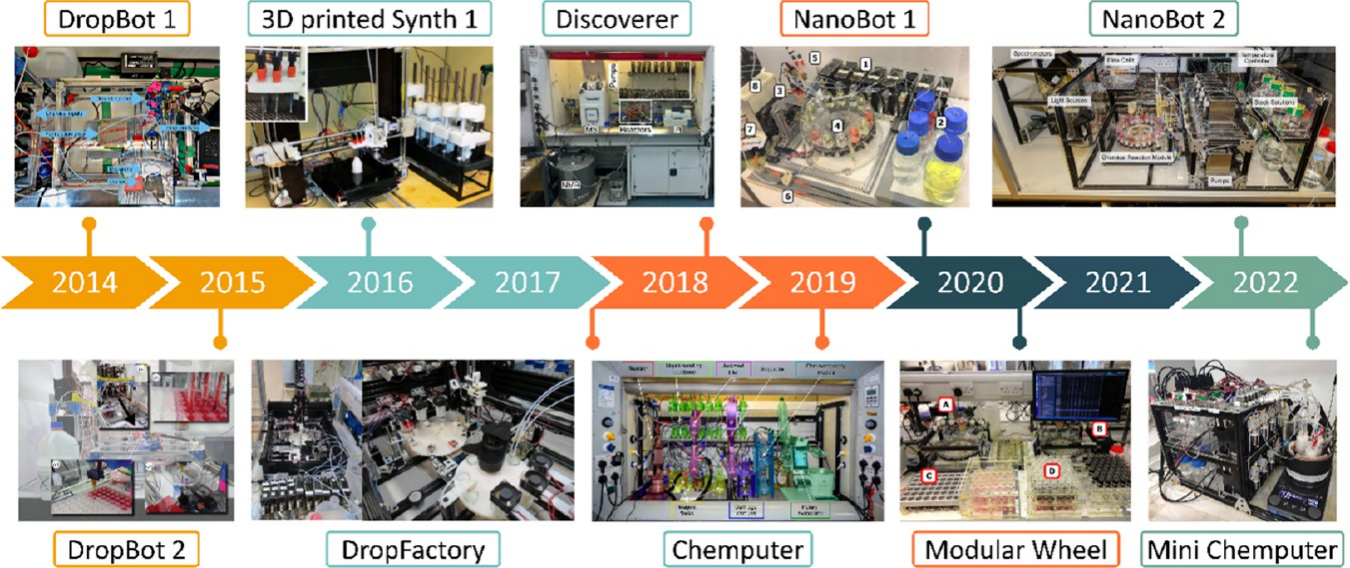
Platform evolution timeline for the most representative platforms developed within the group for discovery, multistep organic/inorganic synthesis, and formulations.

The digitization of chemistry requires the development of a standard architecture for “*chemputation*”: this is the ability to produce a given experimental outcome with a series of chemical inputs within a standard universal “computing” machine for execution of the chemical experimental process. To achieve this, we designed a new ontology for combining unit operations across a range of modular automated chemistry tools. These tools share common operations, i.e., liquid handling, stirring, separation, analysis, etc. During the past decade, we have been exploring the development and use of digital workflows ranging from relatively simple systems for liquid handling, to highly complex platforms capable of performing multistep organic/inorganic synthesis including a dynamic analysis for closed-loop approaches; see Figure 1. Based on their capabilities and intended function, the systems developed within the group can be classified into four categories: (i) *discovery*: platforms that include complex analytical techniques for closed-loop approaches to batch organic reactions in the hope of discovering new reactivity; (ii) *formulation* platforms for the high throughput production of droplets/formulations to study and optimize for specific behaviors; (iii) *batch* platforms capable of performing multistep organic synthesis; and (iv) *modular* platforms for the high throughput screening and/or closed-loop exploration of nanomaterials. Across all four categories, software was a means to an end (i.e., the desire for a system to work for its specific project), resulting in a lack of software continuity, causing a significant loss of time when moving from one system to the next. Over the years this problem was solved by developing unified software packages to control specific systems (e.g., syringe pumps, stepper motors, sensors, etc.). These packages and the system’s architectures were further developed or modified later to converge them into the chemputation framework to be run using a chemical programming language (χDL).^15^ All the aspects of digital chemistry listed above may be achieved using a variety of methods or hardware; however, χDL being a high-level abstraction can provide a shared standard operating software.

Each platform type was being developed within the chemputation roadmap concurrently, so they overlap chronologically; however, each category is described in its own timeline for clarity. N.B. in some cases publishing happened later than when the practical work was completed; thus, each section describes systems development chronologically rather than a publication timeline. Also there have been several examples of standalone projects and automated systems developed in the group for a singular function/idea and do not quite fit into the four central categories. Examples include crystal formation to produce random numbers,^16^ synthesis of biological-inorganic peptide structures,^17^ using machine learning to explore sequence space for antimicrobial peptide discovery,^18^ and pitting humans against algorithms in a competition to discover and crystallize giant polyoxometalates^19,20^ to name a few.

Closed loop systems that can perform chemical reactions, analyze outcomes, and infer from the results the best next step toward a target goal are both the most difficult and most sought-after processes to achieve. For such systems, several needs must be satisfied. First, the hardware capabilities need to match the synthetic, purification, and analysis operations. Second, the analysis method must ideally be fast and provide significant relevant feedback of results. Finally, alongside the operational software, the system must have the means of learning from the results and proposing the next step toward a goal. Many of our systems have achieved this type of workflow, while others demand such significant challenges for achieving it that they have not yet been attempted. However, digital, automated, or “self-driving” systems are tools like any other, and an obsessive pursuit of the perfect closed-loop system can miss this point, and we have found that even the simplest platform can have a profound impact on daily laboratory work.

## Discovery Systems

Chemical exploration is slow, not only because the chemical space is vast but also because many experimental procedures are tedious and not easy to reproduce. Our discovery platforms were designed to explore chemical space in a more efficient way to accelerate the discovery rate. We have developed five versions of these systems to date, each following a similar physical workflow, capable of a closed-loop approach but increasing its synthetic/analytical capabilities from one version to the next. Improving their chemistry accessibility allowed us to explore more complex procedures along with improved algorithmic approaches. These systems used a series of syringe pumps connected in a daisy chain to form a liquid handling backbone architecture. This method connected all areas of the platform to one another using the pumps as a shared conduit and is still used widely within the group today.

**Figure 2:**
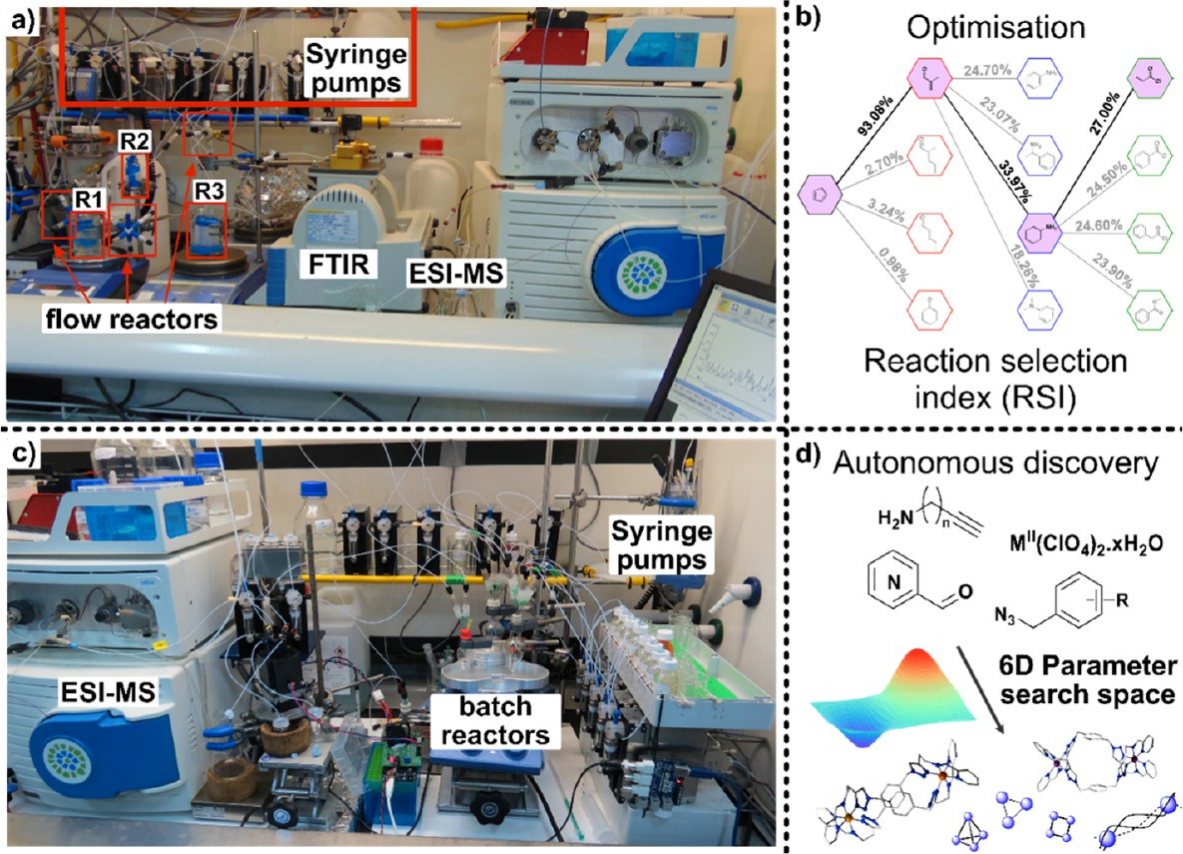
Finder platforms. (a) Three flow reactor platforms used for reaction discovery composed of an ESI-MS and a flow FTIR for analysis, and syringe pumps for liquid handling. (b) Optimization pathway using a reaction selection index (RSI). (c) Inorganic finder platform used for an autonomous discovery of inorganic moieties. The platform is composed of an ESI-MS for analysis and syringe pumps for liquid handling. (d) Helical coordination complexes discovered in a 6D parameter search space.^23^

In 2015 a first version of this system type was developed for the optimization of organic reactions by using a flow reactor, supplied by a series of syringe pumps, with an in-line benchtop NMR to monitor organic reactions in real time.^21^ Initially, this system was controlled via a modular LabView software, while characterization (i.e., NMR), data analysis, and an optimization algorithm were used to complete the closed loop system. This platform was used for real-time structural characterization of reaction mixtures using ^19^F NMR, ^13^C NMR, DEPT NMR, and 2D NMR spectroscopy (i.e., COSY, HSQC, and ^19^F-COSY), along with the optimization of a catalytic organic reaction. This system demonstrated an applicability to self-optimize reactions based on stereoselectivity and multinuclear measurements. Following the success of this simple flow platform, in 2017 we moved to an exploratory platform to search the chemical space using reactivity as the guide on batch reactions, Figure 2a. The platform was updated by combining three flow loops in sequence, in-line spectroscopy (ART-IR and ESI-MS), and an algorithm to differentiate and select the most reactive pathways.^22^ We proposed the use of a reaction selection index (RSI) to allow automated navigation of a defined chemical space. This led us to synthesize previously unreported molecules while performing a fraction of the possible reactions. We demonstrated that RSI can be correlated with reactivity and can search for a defined chemical space using the most reactive pathways. Concurrently to this system in 2017, a similar platform was used for the discovery of supramolecular architectures, Figure 2b.^23^ The system was designed to search for areas of reactivity through an autonomous selection of the reagent types, amounts, and reaction conditions. The reaction solution was then analyzed by evaluating differences in pH, UV–vis, and mass spectra before and after the search was started. This led to the discovery of a range of 1-benzyl-(1,2,3-triazol-4-yl)-*N*-alkyl-(2-pyridinemethanimine) ligands and new complexes: [Fe(L1)_2_](ClO_4_)_2_; [Fe(L2)_2_](ClO_4_)_2_; [Co_2_(L3)_2_](ClO_4_)_4_; [Fe_2_(L3)_2_](ClO_4_)_4_, which were crystallized, and their structures were confirmed by single-crystal X-ray diffraction determination, as well as a range of new supramolecular clusters discovered in solution using high-resolution mass spectrometry. In terms of hardware these two systems were very similar, using the same syringe pumps and general flow set-ups as seen previously; however, they diverge in the analysis techniques which depended on the chemistry, thus proving the versatility of the finder platforms. Both systems were among the first examples of reusable codes for the synthesis of organic molecules.

**Figure 3:**
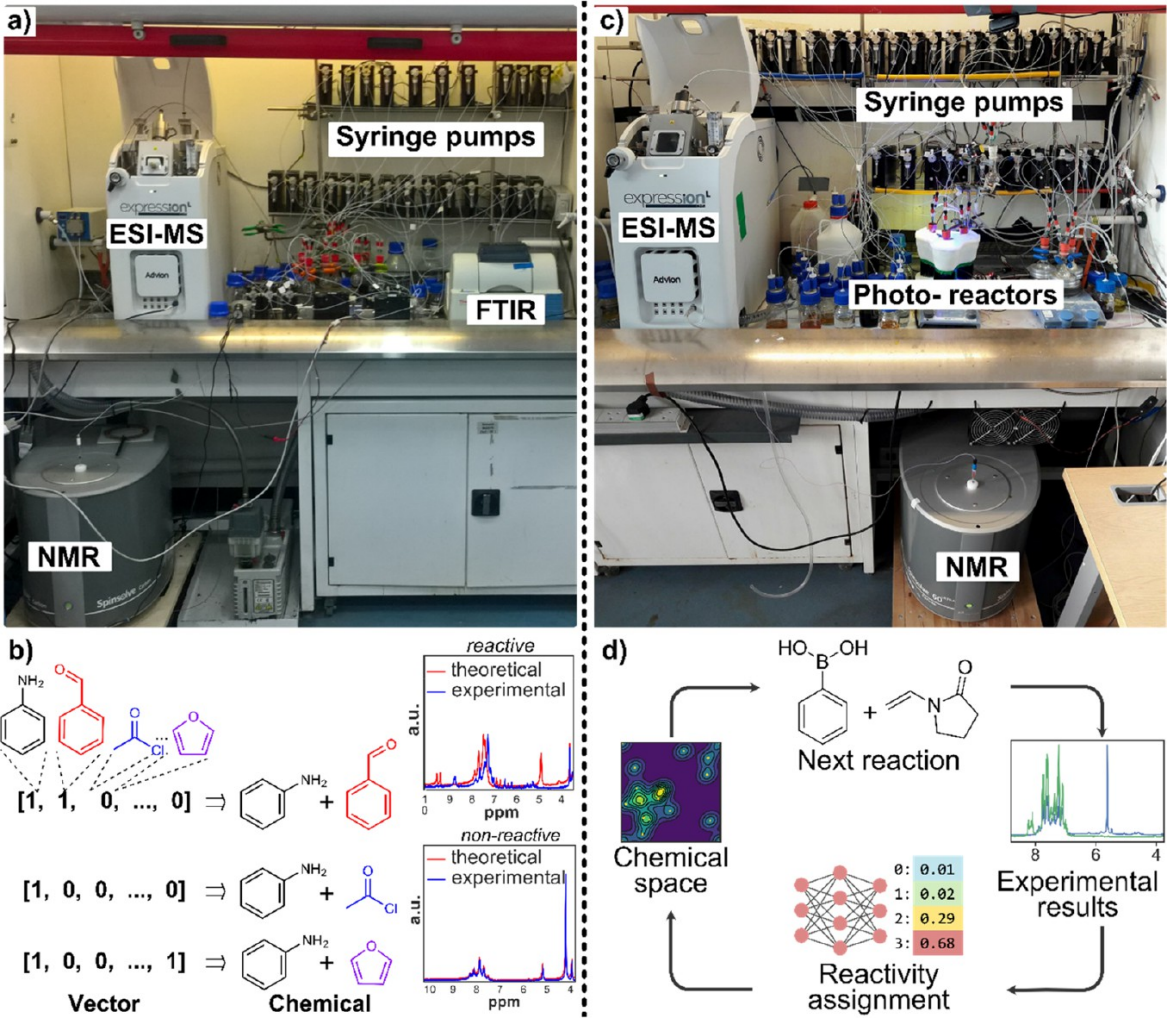
Reactivity prediction finder platforms. (a) Automated platform for reaction prediction comprised by an ESI-MS, NMR, and AT-IR for analysis and syringe pumps for liquid handling. (b) Optimization pathway using the reaction selection index (RSI). (b) Vector representation of chemical reactions used for determining if the mixture was reactive or nonreactive. (c) Photoreactor finder platform composed of an ESI-MS and NMR for analysis and syringe pumps for liquid handling. (d) A closed-loop approach for chemical space exploration where after every reaction and NMR and MS spectra are collected, processed, and used to formulate the next experiment to be performed automatically.^24^

Up to this point, our finder systems relied solely on results from the reactions performed, having limited predictive power when considering how to search for unknown reactions. In 2018 we devised an automated system that was controlled by a machine learning algorithm, which formulates the task as a binary classification problem, to accurately predict reactivity of a set of reagents if trained using a small subset of reactions performed using the automated system, especially if classified by an expert chemist; see Figure 3a.^24^ The system comprised a single mixing vessel charged by a series of commercial syringe pumps. Once the reagents are mixed, a reaction solution was transferred for a set period of time to one of six reactor vessels that worked in parallel, before being analyzed in flow by NMR, MS, and ATR-IR. Initially, the system performed 72 reactions and classified them into reactive or nonreactive manually.

The data were then used to build a series of machine learning models, building upon one another to produce predicted reactivity scores for reactants and reaction conditions. The reactivity of about 1,000 reaction combinations was predicted with an accuracy greater than 86% after analysis of the outcomes of ca. 10% of the data set. These predictions led to the discovery of four new reactions. The fifth, and currently final, version of this system type was a robotic platform capable of navigating a chemical space based on a learned general association between molecular structures and reactivity; see Figure 3b. For this we incorporated a neural network model that could process data from online analytics and assess reactivity without knowing the identity of the reagents.^25^ This new version of the platform included a photoreactor that, in conjunction with this learned knowledge, was able to explore potential reactions and assess the reactivity of mixtures of unknown chemical spaces, regardless of the identity of the starting materials. The validation of the system was done within a budget of 15 inputs combined in 1018 reactions, further analysis of which allowed us to discover not only a new photochemical reaction but also a new reactivity path for a well-known reagent (*p*-toluenesulfonylmethyl isocyanide, TosMIC). This involved the reaction of 6 equiv of TosMIC in a multistep, single-substrate reaction with the formation of five new C–C bonds. An analysis revealed that this transformation was intrinsically unpredictable, demonstrating the possibility of a reactivity-first robotic discovery of unknown reaction methodologies without human input. This is because the overall network is vastly larger in size than the subset potentially leading to a product (10^10^ chemicals vs 10^5^ chemicals), indicating that the observed pathway is highly improbable to predict *a priori*. The five *finder* type automated systems shared many things conceptually; however, they suffer from a significant issue we have encountered in chemistry automation: software continuity. Despite similar hardware set-ups, significant variation was allowed in the control software between systems (e.g., four different software controllers for the syringe pumps alone were used). The final version of the system utilized one of the first cross platform control software packages developed within the group for the control of commercial syringe pumps (see formulation platform section).

## Multistep Organic Synthesis Systems

The automation of multistep organic reactions has been limited to a small set of molecules including oligonucleotides and polypeptides. These syntheses have been facilitated by iterative processes, which in recent years has been expanded to oligosaccharides and small organic molecules.^11,12^ However, this still limits the number of products available for automation to a small set of reactions, leaving a wide range of laboratory- and discovery-scale synthetic procedures predominantly as manual processes. Multistep organic syntheses require several complex processes that can be linearly executed to achieve a target molecule. Each of these processes requires different hardware and software integration, which together can form a complex workflow.

**Figure 4:**
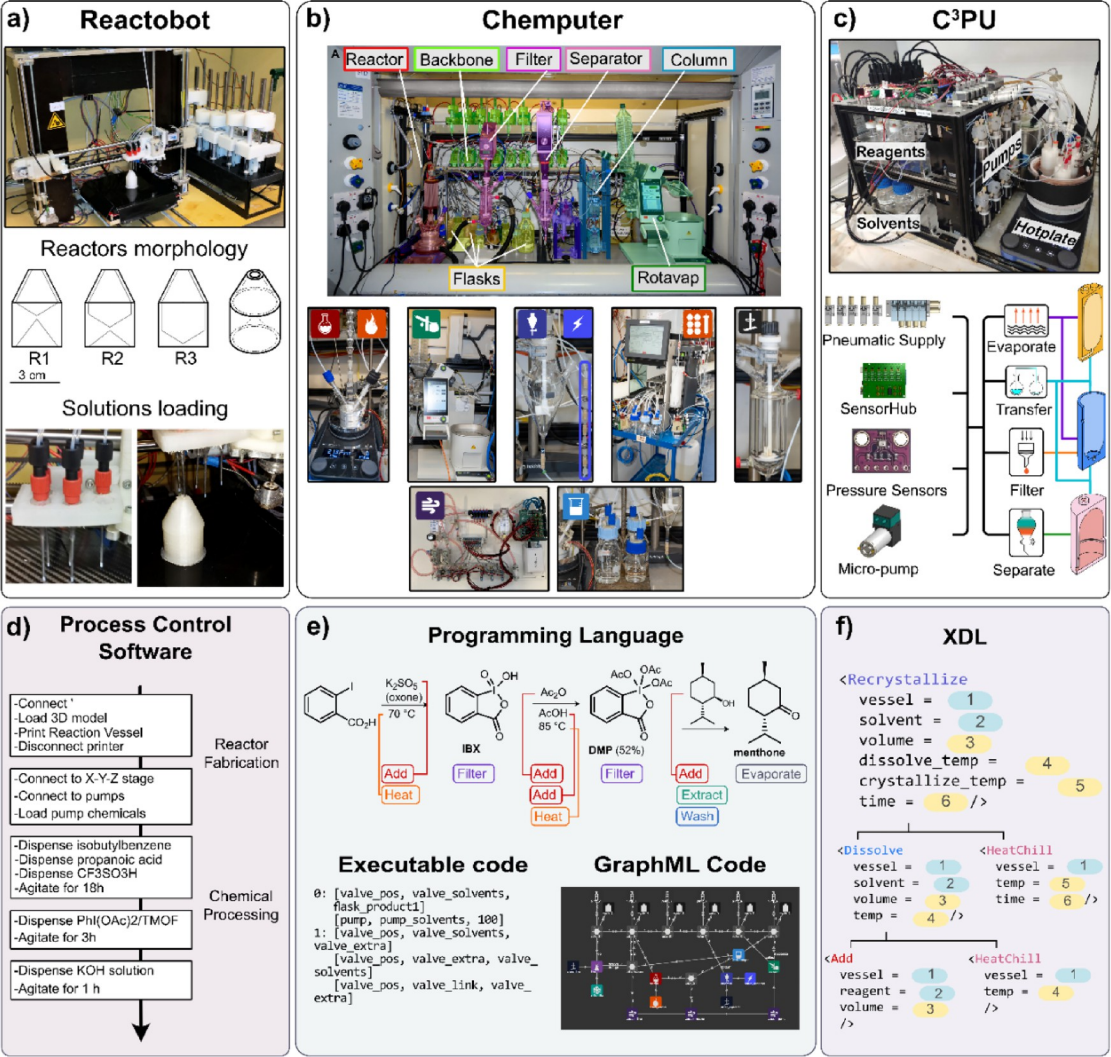
Multistep organic synthesis systems. (a) Reactobot platform: a 3D printer architecture along with in-house built syringe pumps to print *reactionware* vessels and dispense reactants simultaneously used for the synthesis of ibuprofen.^26^ (b) Chemputer platform: an organic synthesis system used for the preparation of diphenhydramine hydrochloride, rufinamide, and sildenafil.^28^ (c) C^3^PU platform: A miniaturized chemputer implementing *reactionware* systems used for the synthesis of small organic molecules, oligopeptides, and oligonucleotides.^38^ (d–f) Software implementation evolution that came along with the platforms, starting with a simple process control software to execute individual steps (d), going through to a programming language that involves higher level execution steps along with a graphical representation of the physical instances of the platform and connectivity (e),^33^ and finalizing with XDL, a platform independent programming description language (f).^31^

This goal started in 2016 with a simpler initial idea, where an automated synthesis robot was built by modifying an open-source 3D printer (Figure 4a).^26,27^ The system was used to 3D print reaction vessels (*reactionware*) of differing internal volumes using polypropylene. The manufactured vessels were subsequently used for the synthesis of the anti-inflammatory drug ibuprofen via a one-pot, three-step approach. The synthesis scale could be adjusted by modifying the parameters in the robot control software. For liquid handling, syringe pumps were developed within the group, while to control the platform operations a python script was written containing the hard coded synthesis of ibuprofen (Figure 4d). This project introduced not only the in-house developed syringe pumps but also the idea of creating and sharing validated synthetic “programs” which can run on similar robotic platforms.

To further develop this concept, in 2019 we introduced the *chemputer* platform, the first system capable of chemputation from the ground up; see Figure 4b. This included an abstraction that maps commonly reported protocols into discrete executable steps.^28^ To implement this concept, six-way valves (also developed by us) were added to the previously used syringe pumps to form a liquid handling backbone. This interconnected backbone represented the core of the platform, as it allowed the movement of solutions to and from any location on the system. To increase accessibility to traditional chemical operations, a conductivity sensor was added to detect phase separation during liquid–liquid extractions. Along with this device, a hot plate and a rotary evaporator also formed part of a standard *chemputer* setup. To control and execute the unit operations, a programming language called χDL (pronounced “Chi” DL – chemical description language) was developed and introduced to formalize and control the assembly of the molecules; see Figure 4e. We validated the concept by synthesizing three pharmaceutical compounds: (i) diphenhydramine hydrochloride, (ii) rufinamide, and (iii) sildenafil. The syntheses protocols were captured as a digital χDL code that can be published, versioned, and transferred enhancing reproducibility and reliable access to complex molecules. This process was the first true instantiation of what we call *chemputation*, i.e., the process of converting the synthesis of molecules to an executable format. Contained within this executable is the platform graph which is a graphical representation of the platforms configuration for a given synthesis, the reagents required, and the sequence of steps to produce the molecule; see Figure 4e.

Although having a platform that can perform multistep organic synthesis constituted a big step toward automation, bespoke hardware configurations were needed depending on the target molecule. This means that the connection of multistep syntheses in a single machine to run many different protocols and reactions was not initially possible, as manual intervention is required. However, we reasoned that converging different reaction classes into a single modular platform can improve chemical accessibility without the need for multiple platforms. To achieve this we showed that the *chemputer* can be programmed to perform many different reactions, including solid-phase peptide synthesis, iterative cross-coupling, Grignard’s reactions and accessing reactive, and unstable diazirines.^29,30^ Developing universal and modular hardware that can be automated using one software system makes a wide variety of batch chemistry processes accessible. Adding a jacketed filter to perform filtrations and reactions over a wide temperature range enabled the synthesis of peptides. We performed around 8,500 unit operations in the *chemputer* while reusing only 22 steps in 10 unique modules, with the code able to access 17 different reactions. Each of modules uses standard unit operations for the steps like “add”, “stir”, “heat”, “chill”, “evaporate”, “separate”, “filter”, and “dry”, which are abstract steps in which each has a different specific implementation bound to the module which affects that abstract operation.

This universality and capability of integrating different chemistries and methods were demonstrated by synthesizing a complex synthesis of a peptide reacted with a diazirine—a process requiring 12 synthetic steps.

After converging the hardware needed into a single and complex platform, we realized that it can be difficult to run and maintain the chemical codes generated from the synthesis, as there was no strict standard defined. The advantages obtained by converging syntheses protocols can be lost if every platform is using independent software. To ensure that the χDL was built on a solid foundation, we aimed to define a software standard that showed that an extendable chemical execution architecture could be generated. This was done by automatically reading the literature, leading to a seamless workflow.^31^ This system was unified in our ChemIDE software^32^ which combined a heuristic for reading a block of synthesis text using NLP and converting it into the χDL code. This is done by pasting in the synthesis text, and the NLP will generate the procedure in steps, the χDL code, and also populate a template graph so the code can be chempiled (like software compilation) and run on the robot. This chemical χDL code, combined with a graphical representation of hardware modules, could be compiled into low-level robotic instructions for execution on any compatible hardware. By running 12 different χDL’s on a six different machines, we showed that the language is hardware independent and process descriptive (Figure 4e).

To show the platforms’ full capability then we began to generate a database of 100 χDLs for molecules that represent a range of reactions found in contemporary organic synthesis.^33^ To expand the accessibility to various chemistries, an automatic purification module based on chromatography was seamlessly coupled to the platform and programmed with the same language. The type of reactions included in the database included transition metal-catalyzed coupling reactions, heterocycle formations, functional group interconversions, and multicomponent reactions. The chemical reaction codes (χDLs) for the reactions can be stored in a database for version control, validation, collaboration, and data mining.^33^ Of these synthetic procedures, more than 50 entries from the database have been downloaded and robotically run in seven modular *chemputers* with yields and purities comparable to manually executed protocols. Full instructions for installing and using the χDL 2.0 standard that ran these synthetic procedures across all platforms is available and open-source.^34^

Recently, we incorporated the concept of *reactionware*, showcased in *reactobot*, with a *chemputer* to design, build, and validate a compact automated platform for multistep syntheses. *Reactionware* systems are a series of discrete modules that are designed to perform sequential synthesis operations to obtain a target molecule.^35−37^ This allows for the miniaturization of the laboratory hardware needed for the synthesis of any organic molecule. These modules can be assembled into single monolithic units accommodating the synthesis in terms of the connectivity, volume needed for the reactions, separations, and crystallizations, and configuration of the reactor for a given synthesis process. To operate the monolith, the platform was designed to have a programmable manifold to control vacuum/gas flow through the monolith, a liquid handling backbone to allow the movement of solutions/solvents from any receptacle in the system to any module in the monolith, and a pressure sensor to control and monitor the operations within the cartridge. This portable system was demonstrated in the synthesis of phenelzine sulfate (an antidepressant drug), isoniazid (an antibiotic drug for tuberculosis), dihydralazine (an antihypertensive drug), lomustine (an alkylating agent used in chemotherapeutic cancer treatments), and umifenovir (an antiviral medication for the treatment of influenza). This was extended to perform iterative solid-phase syntheses of oligopeptides (VGSA, GFSVA, FVSGKA, and SKVFGA) and oligonucleotides (5′-TACGAT, 5′-CTACGT, 5′-GCTACGAT, and 5′-ATGCTACGGCTACGAT). Simultaneously, due to the implementation of pressure sensors, the platform generates a reaction pressure fingerprint used to monitor the reaction processes within the modules and remotely perform quality control. This platform allows the miniaturization of a chemical manufacturing plant into a small-footprint (250 mm × 660 mm × 390 mm) synthesizer.^38^

## Formulation Systems

The *finder* and *chemputer* platforms were specifically designed around a liquid handling backbone architecture to move liquids to static devices for synthetic operations, which implies there are no moving parts beyond single axis movement (e.g., pumps plungers). This architecture works well for performing a range of synthetic procedures on a medium scale but is a poor choice for higher throughput screening (HTS). Droplet chemistry involves a broad range of applications such as reaction miniaturization, small scale cell culturing, and interfacial chemistry.^39−41^ It is one of many areas of interest within the group that required HTS and so required a fundamentally different approach. Our specific interests in developing automated droplet platforms were to look for behavioral novelty at the material scale using computer vision.^42,43^ This was because we were interested in using computer vision to help discover new phenomenon, to help us explore the physical properties and characteristics of the formulation in real time, and for use as a proxy for other more advanced and time-consuming analytics.

**Figure 5:**
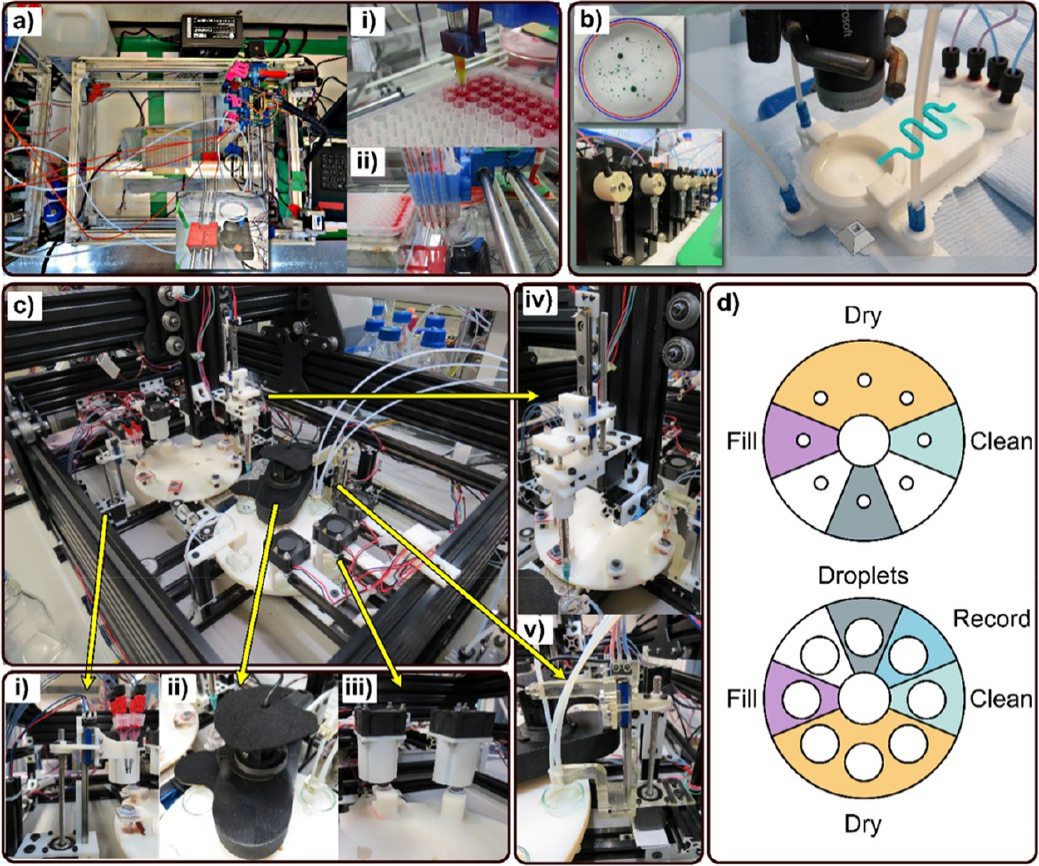
Droplet platforms. (a) Dropbot 1: first droplet robotic, developed from the Rep-Rap 3D printer architecture. (i) Top view of the preparation and moving axis assemblies, (ii) mobile syringe unit preparing droplet mixtures in a well plate, (iii) multiple syringes producing droplets in a Petri dish above a camera setup. (b) 3D printed droplet flow platform. (c) DropFactory system composed of a dual Geneva wheel setup. (i) Droplet material dispensing assembly, (ii) light isolated recording, (iii) drying fan positions, (iv) cleaning station, and (v) assembly for extracting material from the production reservoir to produce droplets on the experimental station. (d) Graphical scheme of Dropfactory showing the action performed at each of the 8 positions of both Geneva wheels.^47^

The droplet systems were our first line of platforms to use moving axes within an autonomous workflow, where syringes, tube assemblies, and reaction vessels could be moved around the platform as required. This series of platforms began with a single focus on droplet chemistry; however, it later led to an explosion of systems within the group for a wide variety of chemistry (see Modular platform section). This was achieved with the development of bespoke hardware setups and the convergence of control software. In 2014 we began to explore the properties of oil droplets as a function of composition via an automated evolutionary process using a liquid handling robot.^44^ The robot was constructed and controlled using the RepRap 3D printer architecture, and it could create droplet mixtures using four different compounds in different ratios in well plates; see Figure 5a, 1–3. The axis moved a syringe assembly to this plate to extract a small volume to create the droplets in a Petri dish containing a surfactant. Once formed, the droplets were recorded using a webcam, and their motion behavior was analyzed using image recognition software based on the OpenCV library. In separate experiments, the fitness function discriminates based on movement, division, and vibration over 21 cycles, giving successive fitness increases. This platform was the first system to use Arduino microcontrollers for operation as well as 3D printed parts for customized assemblies. In 2015, the same system was used again to explore the potential for unconventional computation using droplets.^45^ In 2017 we published a platform that diverged from the typical moving axis designs; however, it is critical to mention, as it further introduced hardware and software capabilities that would influence future projects. The system comprised a 3D printed polypropylene flow setup attached to an arena for droplets observation;^46^ see Figure 5b.

In 2020 we published a robotic platform equipped with a curiosity algorithm (CA) that efficiently explored the states a complex droplet system can exhibit; see Figure 5c, 1–5.^47^ The system used a dual Geneva wheel central assembly, one responsible for the production of oil mixtures and the second being used for the experimentation and imaging (Figure 5d). Droplet material is transferred from production to experimentation by a syringe assembly on an XYZ axis. Each wheel has 8 stations capable of dispensing, cleaning, drying, and extraction distributed around the wheel. On the production side, Eppendorf tubes are used to mix small scale reservoirs of oil mixtures, used to form droplets, and includes a full cleaning apparatus for continuous use. On the experimental and imaging side, each of the 8-wheel positions holds a single Petri dish, each of which is filled with a surfactant before droplets are formed by syringe. The Petri dish is immediately moved to a light isolated position for recording using a HD, high frame rate camera, before moving through a series of cleaning and drying positions. This workflow performs various tasks concurrently and can complete up to 300 experiments in a single day. In and of itself this project was an impressive display of a multiparallel automated closed loop system led by an algorithm; however, its effect internally on the group was far more profound. A short time before this project began, we had purchased a Stratasys Objet Connex 500 3D printer which increased our ability to design, prototype, and build custom assemblies. This was the first platform in the group to make full use of this advanced printing technology. (N.B. this standard of printer has been essential for the rapid *prototyping and development* of many of the systems in our team; however, once complete any of our systems can be reproduced using a range of printers.)

Alongside additional production capabilities, this time was a perfect example of the impact insight from a different but complementary discipline can have to accelerate the progress of a team. Here, by integrating a robotics specialist in the team, many hardware insights provided the group with two critical software packages in the context of robotic control. These have been responsible in large degree for the significant increase in the number of custom assemblies the group has since produced and the speed at which we have been able to create them (see next section). The first package was a library to control all functions of our syringe pumps (Pycont) that unified the software used to control these pumps across all our bespoke platforms. The second library called Commanduino allowed for the direct control of a variety of hardware components through Python, using an Arduino and RAMPs shields. Standardizing the control software removed a significant amount of work and time required for the creation of new assemblies and platforms. Essentially, it allowed us to control stepper motors, sensors, fans, electromagnets, etc. From a hardware perspective, things like design practices, miniature linear systems, a single physical framework, and rigorous standards for platform management (structural hardware, documentation, cable/tube management) were introduced to the group at this time. Individually some of these things may seem trivial, but collectively, they provide versatility and uniformity that have been invaluable in many subsequent projects.

These types of platforms were first designed to replace repetitive and error prone benchwork tasks but rapidly evolved into complex systems for high throughput, algorithm driven experimentation. This happened as ideas for projects involving these systems began to require more complex actions, resulting in the hardware and software advancing to meet the need. Eventually it was clear that, if designed correctly, base modules could be designed and assembled to suit a variety of needs while keeping a standard hardware and software architecture. This resulted in a configurable set of modules that can be assembled into simple to highly capable automated workflows, all controlled via platform independent χDL.

## Modular Platforms

**Figure 6:**
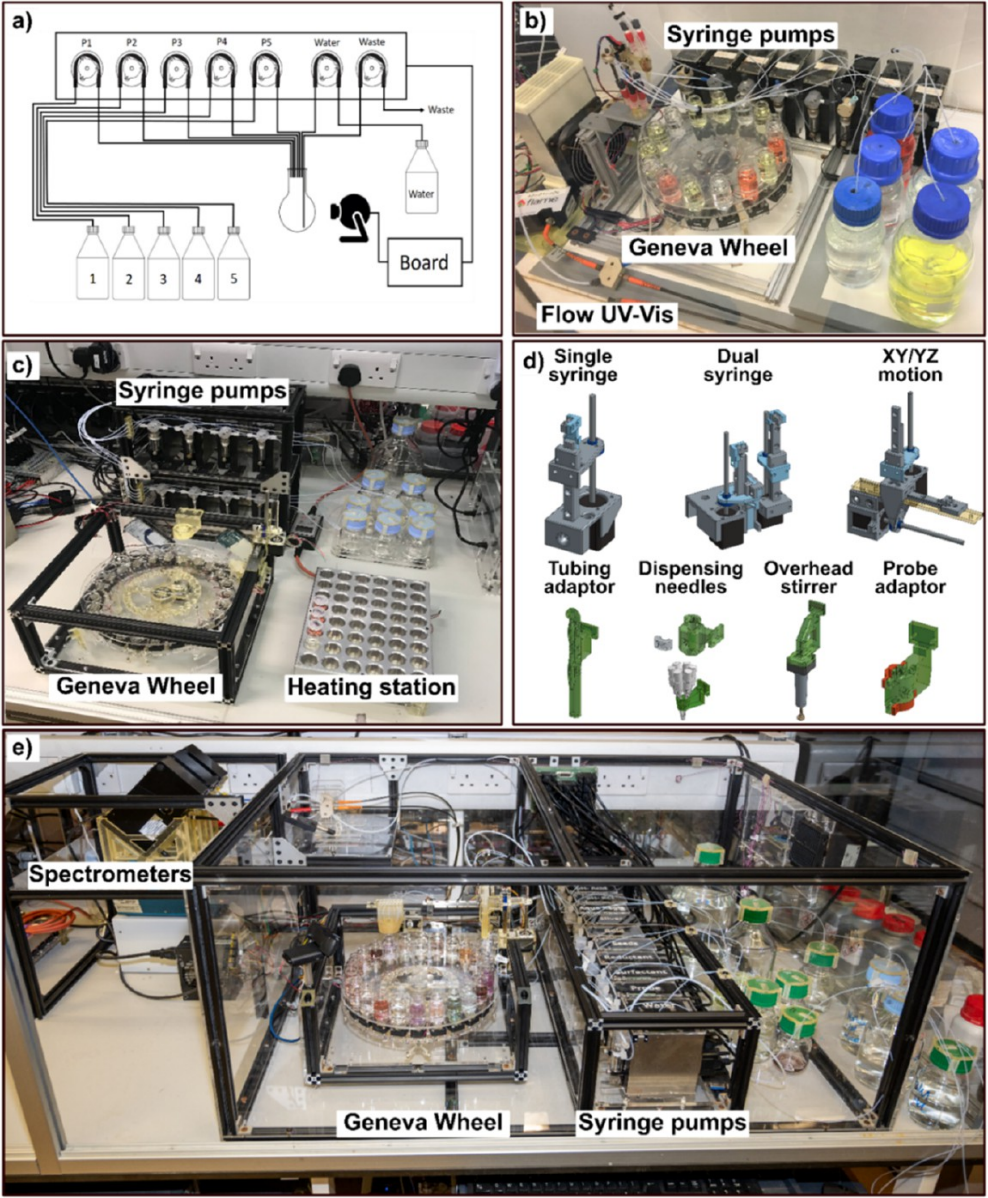
Modular system for parallel syntheses of inorganic materials. (a) Scheme of networked robotic systems used for collaborative synthesis platforms. (b) Nanomaterials optimization platform using a genetic algorithm to target UV signals composed of a 15 vial Geneva wheel with individual stirring and sample extraction assemblies, syringe pumps for liquid handling, and a UV–vis flow setup for analysis. (c) Modular wheel platform (MWP) for high throughput syntheses and exploration of polyoxometalates. (d) The platform is composed of a Z motion and dual Z motion for syringe assemblies as well as an XZ/YZ motion setup. These modules can be used to adapt tubing, probes, dispensing needles, overhead stirring, and electrodes in the automated workflow. (e) Advanced MWP used for the closed-loop multistep nanomaterial synthesis with in-line spectroscopy. The platform includes a pH control, sample extraction, and reaction to reaction seeding assemblies, along with high performance UV–vis, IR and Raman spectrometers for analysis, and syringe pumps for liquid handling.^52^

The first attempt to develop this modular platform was by designing collaborative platforms through social media, via Twitter. Considering the flexibility needed for experimental data collection, the platforms were developed within the group. The systems were simple, consisting of three modules: (i) liquid-handling peristaltic pumps feeding a reactor module, (ii) a camera module for data collection and analysis, and (iii) a control board module. This approach resulted in a simple and affordable (<$500) modular robot with a standard set of hardware and software that can be networked to collaborate in real time; see Figure 6a.

We demonstrated how multiple processes can be done with multiple Internet-connected robots collaboratively, exploring a set of azo-coupling reactions using color recognition, optimizing the crystallization of a known polyoxotungstate using crystal recognition, as well as encoding and decoding information into a network of oscillating reactions.^48^ Conceptually and practically this project was a success; however, on the practical side, the group learned a critical lesson: automation can only be so cheap before significant frustration is experienced. In projects immediately after this we found that with moderate increases in the cost of our control and pump technology, e.g., from PCduino to a standard PC and from aquarium pumps to stepper motor-controlled pumps, respectively, our still cheap and simple systems improved significantly.

In 2018 the first example of a parallel synthesis module was designed and built to perform small scale, batch synthesis of inorganic materials.^49^ This unit was designed to synthesize generations of 15 seed mediated reactions in parallel in a closed loop process for the preparation of gold nanoparticles (AuNPs). The platform consisted of four individual modules: (i) *liquid handling:* commercially available syringe pumps, (ii) *analysis*: flow system for the UV-characterization of nanoparticles, (iii) *reaction module*: a Geneva wheel driven vial tray with individual stirring capability for medium-throughput synthesis, and (iv) *sample extraction*: a small linear motion device designed to extract samples for analysis; see Figure 6b. The system was housed inside a temperature control box and started with raw stock reagents and with no prior knowledge of the chemistry and optimized the synthesis of several AuNP shapes using a genetic algorithm. The design of experiments (DOE) in this system was to perform successive generations of 15 reactions, starting from a random seed. Each reaction of a generation is analyzed by UV–vis and assigned a score based on a spectral target. Crossover and mutation processes of the synthetic conditions of the highest scoring samples were then used to produce the next generation. The cycle continued until the target was reached/sample scoring plateaued. As a demonstration of the reproducibility advantage of automated systems, the highest scoring products from one complete generation series were repeated dozens of times and used as the seeds in the next generation series to form more complex architectures. Over three independent cycles of evolution our autonomous system produced spherical, rods, and octahedral nanoparticles.

The synthesis module described above then evolved into what became known as the *modular wheel platform* (MWP). This system was a refinement of previous versions and was first designed to perform one-pot synthesis of polyoxotungstates for high throughput screening (HTS) and discovery.^50^ The platform consisted of many standalone modules built using a combination of 3D printed, commercially available, and custom-made components; see Figure 5C. To compliment the wheel unit, custom modules for filtration and heating/stirring assemblies were developed to complete the workflow. The complete system could dispense and stir 24 reactions in parallel, heat/stir module 48*n* vials (*n* = number of modules) to up to 150 °C, and a filtration module capable of filtering 48 solutions simultaneously. This system allowed for up to 300 reactions per day for a cost of between £400–1000, depending on the configuration. This digital workflow enabled the discovery of a purely inorganic W_24_Fe^III^-superoxide cluster formed under ambient conditions and the reliable synthesis of the giant W_200_Co_8_ ((C_2_H_8_N)_72_Na_16_ [H_16_Co_8_W_200_O_660_(H_2_O)_40_]) structure, a difficult to isolate molecule, and the discovery of its W_200_Ni_8_ counterpart. This is the first example of a room temperature formed POM-superoxide species without the need for extraneous gas sources. These discoveries were made in a very well-explored chemical space, showing the utility of a simple and cheap HTS system that can allow the researcher to explore a space in much greater detail than could be feasibly done on the bench. The system was controlled using Arduino/RAMPs boards using our in-house Commanduino library developed originally for our droplet systems.

As mentioned above, more complex project demands drove the design of further modules for the system; this began with an external collaboration concerned with creating a system for the formulation chemistry in 2021.^51^ The proposed workflow required multiple layers of probe feedback from the reaction vessels, which then needed to be cleaned to avoid contamination between samples. Miniaturized linear systems for X/Z or Y/Z motion were designed and built to hold turbidity and pH probes and another with conductivity measurement assembly; see Figure 6c.

Up to 8 of these units can fit around the platform frame, accessing any vial position. Each device is cleaned in washing stations built into the corners of the platform base tray. The project used this new functionality for the optimization of formulations by machine learning DoE. The workflow consisted of two such platforms, the first being used for formulation sample preparation and the second for the initial sample analysis. The procedure allowed our collaborators to find nine recipes meeting the customer-defined criteria within 15 working days, outperforming human intuition.

Recently, as a continuation of the previously published system, a platform based around this modular system was used to explore and optimize the multistage synthesis of gold nanoparticles.^52^ This latest system includes a module for the dynamic adjustment of pH and a flow analysis suite for high resolution UV–vis spectroscopy; see Figure 6e. While conceptually similar to the previous work, these systems have a greater synthetic control, and significantly more capable algorithms produced a wide range of particle shapes with unmatched reproducibility. The platform was able to seed nanoparticles of interest from one vessel into a new reactor as well as store samples for later analysis. Most importantly, the system was equipped with a state-of-the-art quality diversity algorithm capable of open-ended exploration and exploitation of the chemistry as well as performing parallel optimization of several particle classes within three hierarchically linked chemical spaces. This process required product confirmation at each synthesis step (analogous NMR/MS analysis in organic synthesis) before continuing with the next step. Using this method, the platform synthesized five distinct AuNPs shapes requiring multistep synthesis in parallel on a single wheel platform in one 24-h period with high yield and monodispersity. Importantly the system was able to output the successful reactions as χDL files allowing the procedures to be run on a range of platforms and be scaled up if needed. Many other projects have been completed or are nearing completion using systems based on this architecture. For example, in 2021/22 the MWP was used as an effective tool for the multistep synthesis of lanthanide-based Mo-POMs with interesting magnetic properties^53^ and for the discovery of several new coordination compounds.^54^ The system’s capabilities continue to increase with each project and iteration.

## Toward Convergence

Over the past decade of the development of systems for digital chemistry and discovery, we have been using a project-based approach to solve individual scientific problems and explore the use of digital technology for scientific discovery. The standardization of hardware and software within the team has recently become a priority for several reasons. To efficiently upgrade and build new systems, having a standard set of hardware modules with a unified software dependent on the same libraries and functions is essential. A common trend in all of the above platform categories was that, at the beginning of automating any new task, the primary goal is to make a working system without much thought for later continuity or knowledge transfer. This is a natural start to any challenge; however, as projects grew in number and complexity, convergence was essential to develop systems efficiently, track progress, and avoid repeating efforts and mistakes. By taking a team-based approach to designing, planning, executing, and analyzing experiments, we have been able to produce many systems for exploring chemical and materials space. In the next phase of the work, we realized that the development of modular systems adhering to a common standard is an extremely useful way to accelerate the design, build, deployment, and gathering of new data. Importantly, we realized that the development of a common standard would be important not only within the team but also for any other researchers to be able to both understand, reproduce, and build upon our results.

**Figure 7:**
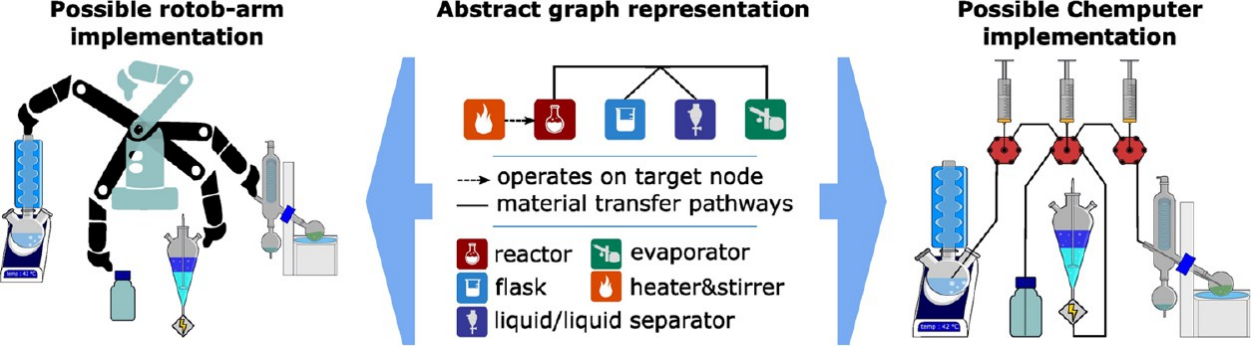
Abstraction of the unit operations for the chemical processes (center) shown applied to a robot arm system (left) and a chemputer system (right).

Platform convergence is important since it should be possible to define in the abstract which unit operations can be achieved by different, but equivalent, hardware. This means that defining the abstract unit operations, specification, chemical compatibility is important for ensuring different hardware can be used to do the same chemical unit operations; see Figure 7.

To produce a proper digital standard that can be adopted by the wider community, we think it is important to adhere to the FAIR principles as for data publishing but applied to software and hardware (Fair: Findability, Accessibility, Interoperability, and Reusability). In our own work, we found that the development of the chemputer and chemputation (i.e., the process of executing the χDL/chemical code on the hardware) could be helpful for many other teams. This is because the chemputer programming language, χDL, is designed to be platform independent, and it uses a naturally emerging ontology that chemists and materials scientists have been using for over two centuries, and hence most of the known literature can be covered.^55^ Chemputation needs the following aspects to be possible: (i) a standard ontology so an abstraction can be built that is programmable; (ii) standard modules that have well-defined specification; (iii) a graph representation of the resources; (iv) actuators to move resources around the modules; (v) sensor systems for feeding back the state of the modules; (vi) standard firmware for interfacing the modules into the application program interface (API) for orchestration of the modules for chempilation (i.e., the process of compiling instructions to be executed on the hardware systems). We feel that convergence of the technologies developed within the group and detailed here will provide an open-source architecture capable of meeting this challenge from high throughput screening, general reaction optimization, and observation to medium scale multistep synthesis.

**Figure 8:**
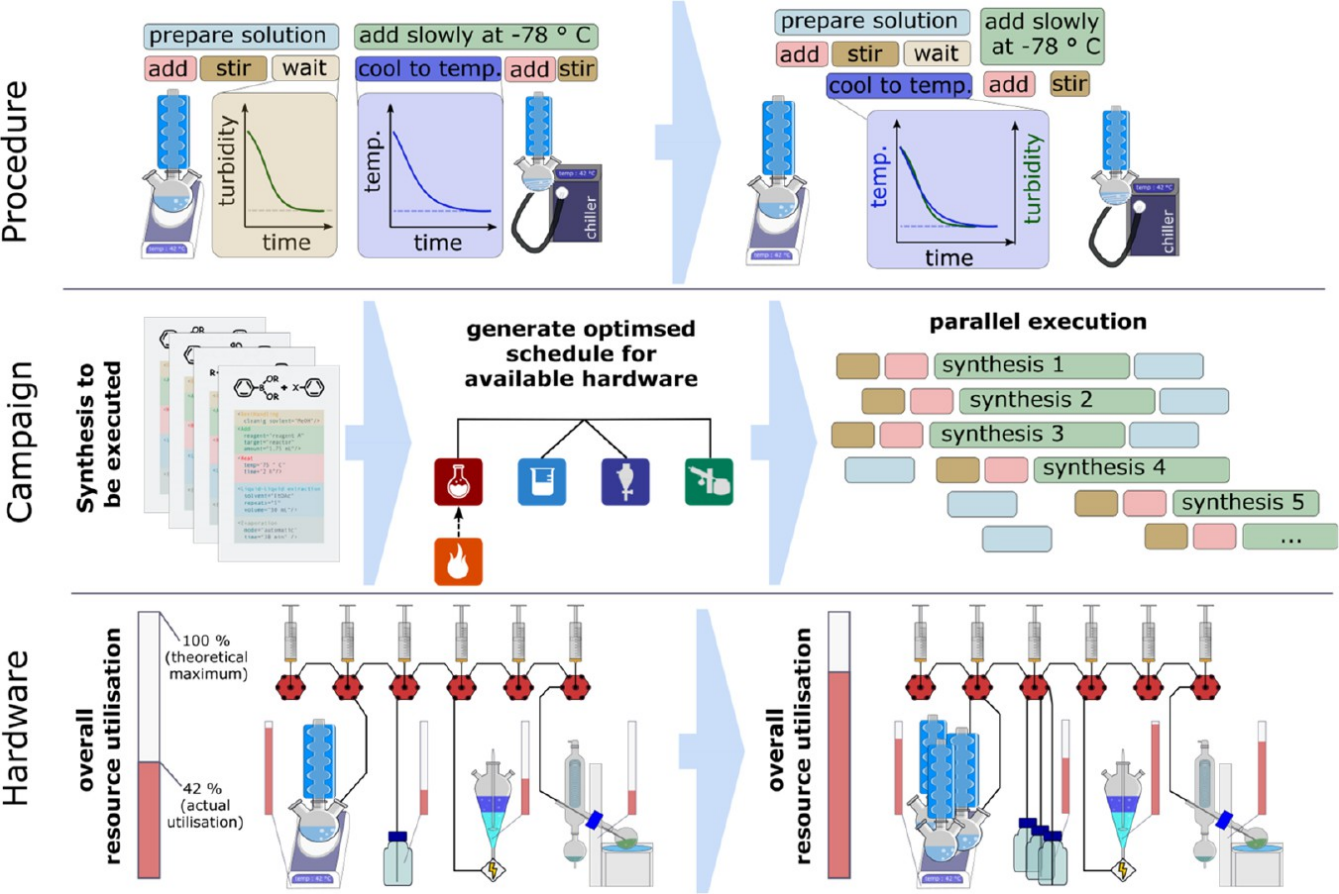
Process optimization from the procedure described in the abstract unit operations to be optimized (top) to the design of experiments campaign (middle) which is then implemented on the hardware (bottom).

Beyond the generalization of the abstraction to different hardware, the use of a programming approach allows a new approach to explore different chemical processes, generating a design of experiments campaign, and then “chempiling” this to a fixed hardware architecture to both optimize and then do the synthesis required; see Figure 8. This means that the optimization processes can be done on several levels: first to explore the process variables to ensure better conversion, selectivity, etc., and second to ensure the hardware is utilized in an efficient manner. The concept of hardware utilization in chemistry and materials science is relatively new because the hardware can be retasked in real time. This can be compared to running programs on multicore processors to more efficiently utilize the hardware to process the computational problems being submitted to the processor.

In the future we are going to be establishing a standards body for the χDL programming language so that the community can help shape and build it further into the common use of the programming language for chemistry, materials, and formulation science as well as biotechnology and molecular biology. This standard abstraction will then be connected to the development of an open standard for modular hardware and software APIs for running the processes. It is our aim that this standard abstraction will be owned by the community and will be integrated with the Open Reaction Database (ORD) and SILA2. Finally, the future of digital matter will be defined by the students and researchers with the aim to accelerate the exploration of new ideas and reproducibility of the scientific literature, but also the discovery of new molecules, reactions, and materials through chemputation.

## Supporting Information Available

Transparent Peer Review report available (PDF)
