# Supplementary material for: Robotic Modules for the Programmable Chemputation of Molecules and Materials
Source: ACS Cent Sci. 2023 Jul 26;9(8):1525–37. doi: 10.1021/acscentsci.3c00304 (PMC10450877; doi:10.1021/acscentsci.3c00304)
Supplement: Supplementary file 1 — oc3c00304_si_001.pdf [file oc3c00304_si_001.pdf]

oc-2023-00304q.R1

Name: Peer Review Information for "Robotic Modules for the Programmable Chemputation of Molecules and Materials"

First Round of Reviewer Comments

Reviewer: 1
Comments to the Author
This Outlook by Cronin and coworkers describes many of their contributions to the space of the digitization of chemistry and their work in developing common software packages and frameworks for control. As this article is meant to express a subjective vision through the lens of the authors' own experiences, my comments will focus on minor suggestions that might improve its clarity and accessibility to readers:

1. When describing the work of Granda et al. [P6L50] it would help to clarify that "accurately predict reactivity" formulates the task as a binary classification problem (rather than quantitative rates, yields, etc.)
2. When describing the TosMIC reaction, providing a small amount of elaboration on the analysis of intrinsic predictability/unpredictability would strengthen the argument about the promise of these discovery platforms
3. Clarifying the precise workflow that is enabled by the Mehr et al. parser [P11L60] rather than describing it as a "universal autonomous workflow" would make this discussion more informative
4. The discussion around reactionware seems less precise in its language than other sections. It is stated that multiple modules are assembled into a "monolithic unit containing all the synthesis information of a specified molecule", but the hardware itself does not contain the information and requires an additional manifold, liquid handler, control/monitoring system, etc., to actually operate.
5. "DOE" and "API" are used without definition
6. I believe many/most of the figures reuse panels from prior publications, but are not cited as such. Having specific references for each panel would help readers follow-up on the individual aspects of greatest interest to them.

Having a greater emphasis on the physical operations each platform requires---throughout the text when each is introduced along with its use cases---might improve the cohesion of the article and the importance of XDL-like standards. In particular, the discussion of the original Chemputer mentions aspects like reusing 22 steps in 10 unique modules; detailing these operations might illustrate why/how they support the many use cases in the paper. There are times throughout when this emphasis comes back up, for example, in the start of the "Formulation systems" section or on Page 16, but the message is sometimes lost when the focus shifts to enumerating contributions of specific papers. The timeline in Figure 1 is helpful for orientation – might a comparable figure highlight hardware/software needs and capabilities as they have converged into a unified language or workflow? I found myself wanting more in the

”Towards Convergence” (e.g., with respect to the ‘requirements’ of chemputation), so to the extent that it is possible to elaborate on this thread, I’m sure other readers would also benefit.

Author's Response to Peer Review Comments:

Reply to Editor and Referee

ABSTRACT: Please make sure the word count does not exceed 200 words, and label as Abstract.

Done and reduced to 200 words

GENERAL REF FORMATTING: Periodical references should contain authors' surnames followed by initials, article title, journal abbreviation, year, volume number, and page range. Refs with more than 10 authors should list the first 10 and then be followed by "et al."

Done

Web sources must include access date.

done

TOC MISSING: Provide a TOC image per journal guidelines (3.25 in. × 1.75 in. (8.25 cm × 4.45 cm) ; on the last page of the Manuscript) with the heading "TOC Graphic" above the graphic. Make sure to designate the file as "Graphic for Manuscript."

Done

SYNOPSIS MISSING: The synopsis should be no more than 200 characters (including spaces) and should reasonably correlate with the TOC graphic. The synopsis is intended to explain the importance of the article to a broader readership across the sciences. Please place your synopsis in the manuscript file after the TOC graphic.

Done

PULL QUOTE (OUTLOOKS + IN FOCUS): We encourage you to select 3 - 4 quotes from your submission that you would like highlighted in your paper. The quotes should be one sentence-long, unique to the submission and not from previously cited work. Please list your quotes at the end of the manuscript file.

Done

Reviewer(s)' Comments to Author:

Reviewer: 1

Recommendation: Publish in ACS Central Science after minor revisions noted.

Comments:
This Outlook by Cronin and coworkers describes many of their contributions to the space of the digitization of chemistry and their work in developing common software packages and frameworks for control. As this article is meant to express a subjective vision through the lens of the authors' own experiences, my comments will focus on minor suggestions that might improve its clarity and accessibility to readers:

1. When describing the work of Granda et al. [P6L50] it would help to clarify that "accurately predict reactivity" formulates the task as a binary classification problem (rather than quantitative rates, yields, etc.)

This has been addressed

2. When describing the TosMIC reaction, providing a small amount of elaboration on the analysis of intrinsic predictability/unpredictability would strengthen the argument about the promise of these discovery platforms

This has been expanded and addressed

3. Clarifying the precise workflow that is enabled by the Mehr et al. parser [P11L60] rather than describing it as a "universal autonomous workflow" would make this discussion more informative

This has been expanded and addressed

4. The discussion around reactionware seems less precise in its language than other sections. It is stated that multiple modules are assembled into a "monolithic unit containing all the synthesis information of a specified molecule", but the hardware itself does not contain the information and requires an additional manifold, liquid handler, control/monitoring system, etc., to actually operate.

This has been expanded and addressed

5. "DOE" and "API" are used without definition

Corrected

6. I believe many/most of the figures reuse panels from prior publications but are not cited as such. Having specific references for each panel would help readers follow-up on the individual aspects of greatest interest to them.

All the figures are new but references to the relevant papers has been added.

Having a greater emphasis on the physical operations each platform requires---throughout the text when each is introduced along with its use cases---might improve the cohesion of the article and the importance of XDL-like standards.

In particular, the discussion of the original Chemputer mentions aspects like reusing 22 steps in 10 unique modules; detailing these operations might illustrate why/how they support the many use cases in the paper.

A section explaining the abstractions has been added to highlight the high-level nature of the operations and that they need to be implemented on the different hardware modules.

There are times throughout when this emphasis comes back up, for example, in the start of the "Formulation systems" section or on Page 16, but the message is sometimes lost when the focus shifts to enumerating contributions of specific papers.

This is an important way the technology evolves. It is a continued process of specific developments for a use case, followed by generalisation and further development. It is important this is explained. In regard to the initial series of multi-axis robotics in the group, these systems were designed and built for specific behavioural studies on formulations originally outside the scope of the version of the XDL standard that existed at the time.

In terms of the detailed operations, page 16 has a list of all the operations the state the of the art system performs and figure 5 gives visuals. As such we feel that further detail would be out of the scope of this article but can be seen in the published work.

The timeline in Figure 1 is helpful for orientation – might a comparable figure highlight hardware/software needs and capabilities as they have converged into a unified language or workflow?

> We agree this is a useful visualization, but again highly detailed figures have been previously published regarding all software architectures used on our platforms.

I found myself wanting more in the ”Towards Convergence” (e.g., with respect to the ‘requirements’ of chemputation), so to the extent that it is possible to elaborate on this thread, I’m sure other readers would also benefit.

> A summary of our vision of a unified system and its capabilities has been added to this section.

oc-2023-00304q.R2

Name: Peer Review Information for "Robotic Modules for the Programmable Chemputation of Molecules and Materials"

Second Round of Reviewer Comments

Reviewer: 1
Comments to the Author
Thank you for considering the recommended changes. I will reiterate my interest in a more explicit discussion of how the hardware/software has converged to common elements, through text or a figure, but recognize that this is subjective and should not be essential for publication

Author's Response to Peer Review Comments:

Reply to referee

Thank you for considering the recommended changes. I will reiterate my interest in a more explicit discussion of how the hardware/software has converged to common elements, through text or a figure, but recognize that this is subjective and should not be essential for publication.

Thanks for your comment. I’ve added two more figures to explain the abstraction first and then secondly the new prospect of resource utilization and the development of new approaches to utilizing the resources now the abstraction levels are high enough.

I like this point, I think it is very important to explain the future resource utilization that will come with the digitization of chemistry.
